# Impact of Carers’ Smoking Status on Childhood Obesity in the Growing up in Ireland Cohort Study

**DOI:** 10.3390/ijerph16152759

**Published:** 2019-08-02

**Authors:** Salome Sunday, Zubair Kabir

**Affiliations:** School of Public Health, University College Cork, T12XF62 Cork, Ireland

**Keywords:** growing up in Ireland, childhood obesity, primary carer, secondary carer

## Abstract

Childhood obesity is a growing concern worldwide. The association between childhood obesity and maternal smoking and/or paternal smoking has been reported. However, few studies have explored the association between childhood obesity and exposure to carers’ smoking status. This study aimed to assess the impact of carers’ smoking status on childhood obesity in a cohort of children enrolled in the Growing up in Ireland (GUI) study. Participants from the GUI infant cohort were categorized into four groups based on their exposure status: Neither caregiver smoked (60.4%), only primary caregiver smoked (13.4%), both caregivers smoked (10.9%). Exposure to primary carers’ smoking (98% are biological mothers) was found to be significantly associated with childhood overweight/obesity at age three (Odds Ratio: 1.30, 95% CI: 1.17–1.46) and at age five (OR: 1.31, 95% CI: 1.16–1.49). Exposure to both carers’ smoking status was significantly associated with increased odds of childhood overweight/obesity across both waves. These findings emphasize the health burden of childhood obesity that may be attributable to maternal smoking postnatally and through early childhood in Ireland.

## 1. Introduction

The issue of childhood obesity has emerged as a serious global epidemic of the 21st century [1]. Recent trends show stabilization of obesity prevalence throughout the population in many nations [2,3,4,5,6,7], including Ireland. However, specific strategies are necessary to reverse the tide towards healthy living [1,8].

The World Health Organization (WHO) reported that the prevalence of overweight and obesity combined for children has increased from 4% to over 18% between 1975 and 2016 [9]. It is estimated that 41 million children under the age of five and over 340 million between the ages of 5–19 are either overweight or obese [9]. Ireland is ranked among the countries with high rates of childhood obesity [10,11]. Current estimates show that about 7% of girls and 6% of boys aged 4–16 are obese in Ireland, putting the country at 58 out of 200 countries in the childhood obesity charts [11]. Interestingly, almost half of the children in Irish households are exposed to secondhand smoke (SHS) [12]. Taken together, both childhood obesity and childhood SHS exposure are important public health challenges for the Irish government.

Childhood obesity is associated with adverse health outcomes throughout the life course, including an increased risk for type 2 diabetes, heart diseases, cancer, as well as lifelong overweight and obesity [13]. Therefore, governmental strategies, for instance, the Healthy Ireland Framework, have adopted a life-course perspective to chronic diseases associated with obesity and other lifestyle factors, such as tobacco smoke exposure [14]. In 2005, the Bogalusa Heart Study showed that obese children between the ages of 6–13 are ten times as likely to become obese adults as those who are underweight or have lower Body Mass Index (BMI) [15]. Worryingly, children living today could be the first generation to live shorter, less healthy lives than their parents [16]. 

Obesity is a complex system, and requires a systems thinking approach to tackle childhood obesity [17]. Clearly, it is evident that effective intervention strategies are necessary to address the modifiable risk factors contributing to childhood obesity [18]. According to the WHO, the increase in childhood obesity can be largely attributed to the changing nature of the environment [19]. Consequently, childhood obesity can only be successfully tackled if we focus on both the child and the child’s prevailing environment [13]. One such environment is the setting where a child spends most of the time, namely, in a household or in a carers’ service. A 2006 survey by the Central Statistics Office in Ireland found that 60% of infants are looked after by a parent/guardian closely followed by 12% looked after by paid carers’ and 11.5 unpaid relatives [20]. However, 98% of the carers’ in our study are biological mothers. Berman and colleagues [21] also found that children who spend more time at home, in the presence of a smoker or living with a carer who smokes have an increased SHS exposure.

Furthermore, SHS exposure has been identified as one of such environmental hazards that are detrimental to the health of the pediatric population [22,23]. Despite a decrease in the prevalence of smoking [24,25], 40% of children aged 3–11 years had SHS exposure at home, and a large proportion of this exposure is recorded in Europe [26,27]. A growing body of evidence indicates that there is a link between maternal smoking during pregnancy (prenatal) and childhood obesity [18,28,29,30,31]. The mechanisms linking this association has been attributed to the presence of psychological mechanism and residual confounding [18,29]. It has also been hypothesized that inhaling the chemicals in tobacco smoke may cause impaired metabolic and immune functions leading to an increase in the child’s susceptibility to obesity [30].

However, there is limited evidence suggesting a link between post-natal or childhood SHS exposure and childhood obesity [32,33]. Using the Danish Birth Cohort, Moller et al. did not find a statistical association as the group with exposure to smoking only postnatally was small (*n* = 140). Furthermore, children being exposed to SHS during their childhood irrespective of their pre-natal or early post-natal SHS exposure has not been well-researched in settings where both childhood obesity and childhood SHS exposure within households are high [32,33]. Ireland is one such unique population setting—one of the highest rates of childhood obesity [11], and also half of the children being exposed to SHS in households [12]. Moreover, the Irish national health strategy has set out targets and goals to tackle chronic diseases associated with lifestyle factors (obesity and tobacco smoke) through a life-course perspective [14,34]. Therefore, this study is timely.

In summary, the carers’ in our study are 98% biological mothers, and it is probable that childhood SHS exposure would primarily be due to maternal smoking. Therefore, the current study has a two-fold rationale; (1) to examine the impact of childhood SHS exposure on childhood obesity risk in a private setting to provide further evidence in support of a healthy living environment, as actioned in the Irish Healthy Ireland strategy; (2) to reproduce similar findings in Ireland that were previously reported in other comparable population settings, using a nationally representative population-based cohort while controlling for potential confounders available to the dataset. 

## 2. Materials 

### 2.1. Participants 

Study children were participants in a nationally representative follow-up study of children residing in the Republic of Ireland (ROI), the GUI cohort Study. A full description of the GUI cohort study design and data collection methods have been described in detail elsewhere [35]. Briefly, 11,134 infants born between December 2007 and May 2008 and their primary and secondary carers’ were recruited from the Child Benefit Register for the ROI using a simple systematic sampling technique [35]. The infants were aged nine months at the start of the study and data collection, three years during the second wave (*n* = 9703), five years at the third wave (*n* = 9001) and 7/8 years at the fourth wave. 

### 2.2. Data Collection Procedures 

This study used data collected in the first three waves of the study. Information was gathered from biological parents, adoptive parents, caregivers, non-resident parents, grandparents, relatives and unrelated guardian where applicable, of the study child via questionnaire-based interviewing and measurements were taken for the carers’ and the children. Household interviews were administered by trained interviewers via Computer Assisted Personal Interviewing (CAPI), and Computer Assisted Sensitive Interview (CASI) for sensitive questions. The response rates were 64% in wave 1, 91.2% in wave 2, and 91% in wave 3 [35,36,37]. Carers’ were separated into a primary and secondary carer. The primary carer was defined as the person who knew more about the study child who was the child’s biological mother in 98% of cases, and the secondary carer was the spouse or partner of the Primary Carer (usually the child’s father or father figure) [35]. Therefore, in this study, we only present findings related to primary carers’, data on secondary carers’ will be available on request. Carers’ completed a detailed questionnaire that provided information on SHS exposure and relevant covariates, including socio-demographics/household information, carers’ relationship to the child, prenatal care and infant’s health and physical developments. The data were re-weighted to account for sampling errors and differences in non-responsiveness. 

The present study received ethical approval from the Social Research Ethics Committee (SREC) of the School of Public Health, University College, Cork (UCC). The GUI cohort study, including the materials and procedures adopted at all stages of the study received ethical approval from an independent Research Ethics Committee convened by the Department of Health, Ireland [35]. 

### 2.3. Outcome Measures: Childhood Obesity 

The main outcome of interest was childhood obesity determined using Body Mass Index (BMI) [weight in kilograms divided by height in square meters]. Weight (to the nearest kilogram) and height to the nearest centimeter) were measured at baseline and at each wave of the study by trained interviewers. Infants’ weight was recorded using a medically approved Class III SECA 835 portable electronic scales to the nearest 0.5 kg. For infant and child height measurements recorded to the nearest millimeter, SECA 210 measuring mat and Leicester height stick were used [35]. All scales had an upper capacity of 50 kg and were graduated in 20 g increments below 20 kg and in 50 g increments above 20 kg [38]. The World Obesity Federation cut-off points for gender and age-specific BMI were used to define and classify overweight and obesity as non-overweight, overweight or obese [39,40].

### 2.4. Exposure: Carers’ Smoking Status 

Carers’ smoking status was assessed at each wave of the study. Primary and secondary carers’ were asked separately by trained interviewers, “Do you currently smoke daily, occasionally or not at all?”. Their responses were aggregated into Yes (daily/occasionally) and No (Not at all). Carers’ smoking status were also categorized into three groups based on their responses (a) neither carer smoked (b) only the primary carer smoked, (c) both carers’ smoked. 

### 2.5. Potential Confounders 

**Figure 1:**
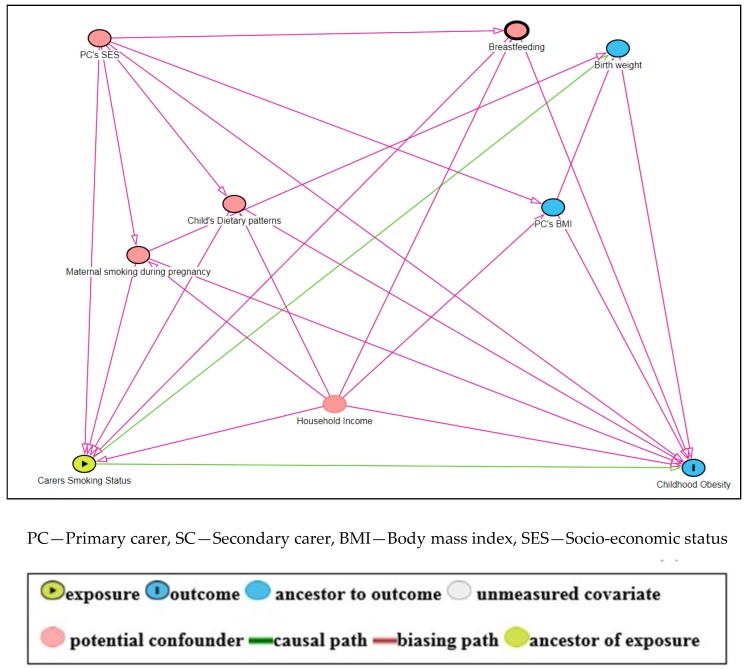
A Directed Acyclic Graph (DAG) for the association between Carers’ Smoking Status and Childhood Obesity.

Directed Acyclic Graphs (DAGs) were used to examine the role of potentially confounding variables of the association between carers’ smoking and childhood overweight/obesity (see Figure 1). The minimum sufficient adjustment sets for estimating the total effect of carers’ smoking on Childhood Obesity was breastfeeding, child’s dietary patterns, equivalized household income, maternal smoking during pregnancy and primary carers’ socioeconomic status (SES). These variables were collected from self-report by the respondent (usually the primary carer). We were unable to control for maternal smoking during pregnancy identified as a strong confounder in this association through the causal diagram (DAGS), because this information was not available. Birth weight evaluated as a categorical variable (<2500 g/≥2500 g) was used instead as a proxy measure of maternal smoking during pregnancy [41,42]. Carers’ socioeconomic status was categorized as “school/education, at work/training, unemployed, home duties, other” as recorded in the questionnaire. Carers’ also reported household income, which was measured by dividing disposable household income by equivalized household size and was represented in quintiles. Breastfeeding was assessed from the question “Was baby ever breastfed?” and was represented as a dichotomous variable.

At subsequent follow up (three and five years), carers’ were asked about the child’s food and drink intake over the last 24 h and month respectively. Three ‘risk-related’ foods were chosen to describe the dietary patterns in children; Fizzy drinks/minerals/cordial/squash (not diet), sweets and hot chips or French fries classified in categories: Yes and No. The choice of risk-related foods is similar to what has been previously described [32]. 

We did not adjust for alcohol consumption and primary carers’ BMI, because our DAG did not identify them as potential confounders. 

### 2.6. Statistical Analysis 

Baseline characteristics of infants and their primary carers’ smoking status were explored using descriptive statistics (mean and percentages were appropriate). Group differences were compared using the independent student’s *t*-test for continuous variables and chi-square test for categorical variables as appropriate. At each follow-up, carers’ smoking status, as well as the proportion of children with normal and overweight/obese BMI, were also summarized using descriptive statistics. 

Crude and adjusted odds ratios (ORs) and 95% confidence intervals (CI) were estimated using logistic regression models to adjust for potential confounders and to estimate the effect between primary and secondary carers’ smoking status separately and children’s BMI in wave 2 and 3 when the children were three and five years of age, respectively. Already defined potential confounders were entered block-wise starting with (i) breastfeeding (ii) Birth weight (iii) Household Income (iv) dietary patterns and (v) carers’ socio economic status. Additionally, we calculated a logistic model for obesity combining both carers’ smoking status as exposures. 

We also compared children who were never exposed to carers’ smoking to (i) Children who were exposed to only primary carers’ smoking (ii) Children who were exposed to only secondary carers’ smoking and (iii) children who were exposed to both carers’ smoking using logistic regression and Mantel Haenszel statistics. Data on secondary carers’ smoking in both waves are available on request.

All statistical analysis was performed using Stata 13 (Stata Corp LP, College Station, TX, USA). A two-tailed P-value, less than 0.05 significance threshold, was chosen for all tests, and R^2^ was used to access the goodness of fit. The directed acyclic graph (DAG) was constructed using a web-based causal diagram DAGitty^®^ version 3.0 [43].

## 3. Results

### 3.1. Overview of Infant and Carers’ Characteristics and Smoking Status 

The distribution of baseline characteristics of study participants (at nine months) and their families stratified by carers’ smoking are presented in Table 1. The analysis showed that the majority of the infants (60.5%) were never exposed to either carers’ smoking, whereas 10.9% of infants were exposed to both carers’ smoking. Conversely, 13.4% were exposed to only primary carers’ smoking (98% are biological mothers). Table 1 also shows that compared to non-smoking carers’, primary carers’ who reported smoking proportionately breastfeed their babies less. Overall, smoking carers’ were more likely to have a household income in the lowest quintile, be unemployed and consumed alcohol weekly.

The distribution of primary carers’ smoking and the prevalence of overweight/obesity in each wave are shown in the Appendix A (Table A1, Table A2 and Table A3). The proportion of primary carers’ who smoked was marginally higher in wave 2 (26.4%) compared to wave 1 (24.3%) and wave 3 (23.4%). Similarly, the highest prevalence of overweight/obesity was observed in wave 2 (23.7%). Due to the low number of children in the obese category, this category was merged with overweight to increase statistical power.

### 3.2. Obesity Estimates in Children at Age Three (Wave 2) and at Age Five (Wave 3) 

Table 2 shows that children exposed to primary carers’ smoking in early childhood had 1.30 times the odds of being overweight/obese at age three compared to children of non-smoking mothers (OR:1.30, 95% CI: 1.17–1.46)). Similarly, children exposed to primary carers’ smoking in early childhood had similarly increased odds of being overweight/obese at age five compared to children of non-smoking mothers (OR: 1.31, 95% CI: 1.16–1.49). 

## 4. Discussion

Given that 98% of primary carers’ in this study were biological mothers, this large cohort study in Ireland showed increased odds of obesity/overweight in children at both age three and five years, if exposed to maternal smoking in early childhood compared with children of non-smoking mothers. Our study findings also suggest that the risk of childhood overweight/obesity following childhood SHS exposure was independent of both low birth-weight and breastfeeding. Both these findings are in agreement with previous studies in Denmark and in Germany [32,33]. However, the Danish Birth Cohort study did not show a statistical association as the group with exposure to smoking only postnatally was small (*n* = 140), and also focused on both prenatal and early post-natal. Our exposed group was larger than the Danish cohort, and, thus, had more statistical power, and also showed that such an effect could continue for a longer period post-nasally through early childhood. Raum et al. also found a positive association between exposure to maternal smoking in the child’s first year and childhood overweight at age six [44]. In short, our study findings are consistent with previous evidence, and were reproducible in an Irish setting, which has local policy implications. 

Considering that the carers in this study were overwhelmingly biological mothers, we can be prudent in stating that the effects, thus observed can broadly be associated with parental smoking. However, in settings where the primary carers are not overwhelmingly biological mothers, there may be variations in the effect estimates because of potentially different underlying mechanistic pathways. Therefore, it is clearly important that similar studies are being undertaken where the carers’ profile varies. However, a growing number of studies have examined the impact of parental smoking postnatally on overweight/obesity in childhood across different population settings, and our observations are in agreement with the majority of these studies [28,32,33,45,46]. For instance, childhood SHS exposure had an increased BMI in these children compared with children who were not exposed to SHS from parents during early childhood [31]. Another study also found a dose-dependent association between exposure to SHS and obesity [47]. A systematic review further validated the relationship between SHS exposure during childhood and increased BMI [18]. The prospective PIAMA Study in the Netherlands showed that ‘smoking in the parental house” was a significant independent predictor of childhood overweight rather than maternal smoking during pregnancy [48].

Not surprisingly, our study failed to show a significant association between paternal smoking and childhood overweight/obesity because of fewer numbers. However, hour study findings are consistent with the Nurses’ Health Study II, where an association was observed for maternal smoking and childhood obesity in the daughter, but not for paternal smoking [49]. Similarly, in the generation R study, in comparison with non-smoking mothers, children whose mothers smoked during pregnancy had increased risk of overweight and obesity at four years old [50]. However, no association was observed for paternal smoking and childhood obesity among non-smoking mothers [50]. Furthermore, the overall significant, but relatively low estimates observed in this study could be attributed to the low prevalence of smoking among carers’ which were much smaller compared to other studies (an average of 24.7% among primary carers’ across all three waves). This can be explained in terms of the increase in smoking cessation campaigns, as well as the comprehensive smoke-free policy in Ireland since 2004 [51]. In addition, parents and carers are more aware of the detrimental effects of exposure to tobacco smoke on children, who are the captive audience. 

### 4.1. Strengths 

To the best of our knowledge, this study is among the first in Ireland to examine the impact of SHS exposure during early childhood through carers’ smoking status (mostly biological mothers smoking postnatally) on childhood obesity using prospectively collected information from a large nationally representative cohort of Irish children. This makes the study generalizable to other comparable population settings. The use of a prospective cohort study minimized the possibility of recall bias from carers’ report of smoking. Additionally, weight and height measurements in the first three waves were taken by trained interviewers, thereby reducing the possibility of recall and interviewer biases (sub-types of measurement bias). Furthermore, important covariates that are not often available in other studies, such as dietary patterns of the children were collected in the GUI Cohort study. We also showed that low birth weight and breastfeeding as individual covariates did not influence the association between childhood SHS exposure and the development of childhood obesity. In other words, low birth weight and breastfeeding can act both as an effect modifier, and a mediator of the association studied. Moreover, the influence may depend on the timing of smoking while breastfeeding and whether the mother inhales nicotine or not. However, for carers who are not biological mothers, the underlying mechanism may be far more complex. 

### 4.2. Limitations 

A primary limitation of this study is that the exposure was based on self-report and no biomarker, such as cotinine measurement was used to verify the exposure. Although some studies have validated the accuracy of self-reported smoking in large populations as an indicator of current smoking behavior [52,53,54], there is still a possibility of under-reporting of smoking status, due to social desirability. This may have led to non-differential misclassification bias, thereby attenuating observed estimates. Similarly, this study did not adjust for exposure from other household smokers. Secondly, this study did not control for maternal smoking during pregnancy and other prenatal exposures because the information was not available, introducing residual confounding. However, studies have shown increased smoking cessation during pregnancy, due to smoking cessation campaigns [55], which has encouraged mothers to quit during pregnancy. Additionally, low birth weight was used instead as a proxy measure for maternal smoking [42]. A similar limitation is that environmental smoke exposure by household smokers and smoke exposure outside the home (e.g., in cars) was not considered as a source of SHS exposure in this study. Nevertheless, the estimates in this study are conservative. 

Although this study set out to examine primary and secondary carers smoking status (this includes biological parents, adoptive parents, caregivers, non-resident parents, grandparents, relatives and unrelated guardian where applicable, of the study child), in the GUI data, 98% of respondents were the biological mother/primary carer), therefore only information on the primary carer was presented. In other words, the study exposure is primarily, due to maternal smoking post-natally.

Additionally, the observed associations might be as a result of residual confounding by unmeasured confounders. For example, this study did not measure factors, such as physical activity which is strongly correlated with childhood obesity [49]. This is an important limitation of this study also identified in previous studies. 

The loss to follow up bias (<5%) is another inevitable limitation of a cohort study that was present in our study. Finally, the possibility of either residual or unmeasured confounding, due to lifestyle, genetic or familial factors [18] and chance should not be ruled out as this may have influenced the findings. 

### 4.3. Future Recommendations and Policy Implications

Future studies should use a reliable biomarker, such as cotinine levels in hair or urine for objective measurement of second-hand smoking, and also capture trajectories across the life-course, with better study designs. Similarly, fat distribution or visceral obesity should be used to determine body fat/obesity in children to account for differences that exist between BMI and body fat distribution, employing objective measurements [56]. 

Nevertheless, our study findings are crucial for supporting the existing body of evidence. The study findings also inform policymakers, government and other stakeholders to refocus on cost-effective strategies (anti-smoking interventions and smoking cessation programs) to tackle childhood obesity in Ireland. Such rethinking among the policymakers will certainly contribute further to the design and evaluation of appropriate health policies and community-based childhood obesity prevention programs in Ireland.

## 5. Conclusions

Both childhood obesity and SHS childhood exposure are public health issues in Ireland. The present study shows that an association does exist between carers’ smoking (largely maternal smoking post-natally) and childhood obesity in children aged three and five years old, independent of other potential confounders, such as low birth weight and breastfeeding. Therefore, this evidence strongly supports the need for informing policies on targeted population-level anti-smoking interventions in private settings not only to reduce further childhood SHS exposure levels, but also to tackle the epidemic of childhood obesity. Such strategies may be cost-effective. Clearly, voluntary smoke-free households need to be strongly encouraged and vigorously promoted.

## Figures and Tables

**Table 1:** Baseline distribution of infant, carers’ and household characteristics according to carers’ smoking status at baseline.

Variables		Neither Carer Smoked	Only Primary Carer Smoked	Both Carer Smoked	*P*
		*n* = 6037 (%) ^a^	*n* = 1339 (%) ^b^	*n* = 1084 (%) ^c^	
Prevalence		60.5%	13.4%	10.9%	
Infant Characteristics					
Gender	Boy	3082 (51.1)	691 (51.6)	558 (51.5)	0.973
	Girl	2955 (49.0)	648 (48.4)	526 (48.5)	
Birth weight	<2500	324 (5.4)	95 (7.1)	75 (6.9)	0.011
	≥2500	5713 (94.6)	1244 (92.9)	1009 (93.1)	
BMI		18.3 (2.7)	18.2 (2.70)	18.1 ± 2.8	
Breastfed	Yes No	4033 (66.8)	521 (38.9)	537 (49.5)	<0.001
		2002 (33.2)	818 (61.1)	547 (50.5)	
Primary Carers’ Characteristics					
Mean Age (years)		32 ± 5.0	28 ± 6.0	30 ± 5.5	
BMI	Healthy	3051 (52.9)	614 (48.1)	514 (49.1)	<0.001
	Overweight	1731 (30.0)	360 (28.2)	320 (30.6)	
	Obese	872 (15.1)	236 (18.5)	166 (15.9)	
Economic status	Pre-school	0	0	0	<0.001
	School/Education	96 (1.6)	37 (2.8)	14 (1.3)	
	At work/training	3667 (60.7)	569 (42.5)	590 (54.4)	
	Unemployed	181 (3.0)	97 (7.2)	48 (4.4)	
	Home duties	2035 (33.7)	619 (46.2)	416 (38.4)	
	Other	56 (0.9)	17 (1.3)	13 (1.2)	

^a^ Also includes those subjects who had lone parents and did not report smoking. ^b^ Contains those whom only primary carer smoked and those who had lone parents, and primary carer reported smoking ^c^ excluded those who reported don’t know or were not present.

**Table 2:** * Adjusted odds ratios (ORs) (95% CI) for the impact of primary carers’ smoking at both waves.

**Primary Carers’ Smoking at Three Years**	**Risk of Overweight or Obesity at Three Years**		
**(yes/no)**	**OR**	**95% CI**	***P***
Unadjusted Model	1.37	1.24, 1.52	0.00
Adjusted Model *	1.30	1.17, 1.46	0.00
**Primary Carers’ Smoking at Five years**	**Risk of Overweight or Obesity at Five Years**		
**(yes/no)**	**OR**	**95% CI**	***P***
Unadjusted Model	1.36	1.21, 1.53	0.00
Adjusted Model **	1.31	1.16, 1.49	0.00

* Reference is non-smoking primary carers’. ** Adjusted for breastfeeding, birth weight, household income, child’s dietary habits and primary carers’ socioeconomic status.

## Appendix A

**Table A1:** Primary Carers’ smoking status at each wave (Wave 1 and wave 2).

Primary Carers’ Smoking	Wave 2 (9793)	Wave 3 (9001)
Yes	2558 (26.4%)	2069 (23.4%)
No	7149 (73.6%)	6784 (76.6%)

**Table A2:** Proportion of children with normal, overweight and obese BMI at each wave (Wave 2 and Wave 3).

	Prevalence of Overweight/Obesity	
	Wave 2	Wave 3
Normal	7267 (65.3%)	7066 (79.7%)
Overweight	1752 (15.7%)	1353 (15.3%)
Obese	514 (4.6%)	445 (5.0%)

**Table A3:** Crude ORs (95% CI) for the impact of primary carers’ smoking at each wave with reference to non-smoking carers’.

**Carers’ Smoking at Three Years**	**Risk of Overweight or Obesity at Three Years**		
	**OR (95%CI)**	**95% CI**	***p***
Primary Carer Smoking	1.47	1.23, 1.76	0.00
**Carers’ Smoking at Five Years**	**Risk of Overweight or Obesity at Five Years**		
	**OR (95%CI)**	**95% CI**	***p***
Primary Carer Smoking	1.48	1.22, 1.82	0.00
